# 

**Published:** 1814-09

**Authors:** 


### MONTHLY CATALOGUE OF MEDICAL BOOKS.

FACTS and Observations (deduced from long and extensive Practice) on Liver Complaints and Bilious Disorders in general; and on such Derangements of these Organs as influence the Biliary Secretions; with some new and practical Observations on the various Appearances of this important Secretion; connected by an appropriate and successful Mode of Treatment, and the whole illustrated and confirmed by a numerous List of Cases. By John Faithhorn, formerly Surgeon in the Honorable East-India Company's Service.—8vo. 5s.

Observations on Pulmonary Consumption. By Henry Herbert Southey, M.D. 8vo. 7s.

Some Inquiries into the Effects of Fermented Liquors. By a Water Drinker. 8vo. Plates, 10s. 6d.

Traité des Poisons tirés des règens Minèral, Végétal, et Animal, ou Toxicologie Générale, considérée sous les rapports de la Physiologie, de la Pathologie et de la Médecine légale. Par M. P. Orfila, M.D. 14s.

---

*Books on Medicine published in Sicily between* 1800 *and* 1812.

Program of a System of Theoretic Medicine, arranged according to the Analytical Method; by Dr. Rosario Scuderi; Palermo, 1804.

Dissertation on the Extirpation of Cancers; by Dr. Franc. Cannizaro di Vizzini; Palermo, 1801.

Advice to Physicians respecting the System of Brown; by Dr. Trusso; Palermo, 1806.

On the Reforms requisite in the Medical Chairs of the University of Palermo; by Dr. Vincenzo Genuardo; Palermo, 1809.

An Historical Essay on the Yellow Fever of America; by Dr. Sciofessie, translated by Dr. Coco; Palermo, 1805.

Medical Considerations and Meditations on Living Man; by Dr. ———; translated by Dr. Carmelo Maravigna, with notes; Catania, 1810.

Essay on Misfortune and its Medicinal Virtues; by Sign. Comandante Poli; Palermo, 1811.

Essay on the Cow-Pox; by Dr. Francesco Calcagni, Physician of the Spedale Grande; to serve for advice to the people and instructions to young physicians and surgeons to practise or direct inoculation, and preserve the matter; Palermo, 1809.

Essay on the Cow-Pox found in the Bocca di Falco, a village near Palermo, in the month of April, 1811; by Dr. Giov. Battista Amati; Palermo, 1811.

---

### NOTICES TO CORRESPONDENTS.

*We are indebted to Mr. Burroughs for an Account of an Inflammatory Fever which prevailed in the 24th Regiment, while in Portugal; also to Mr. Yeatman, for his valuable Communication, which, with others unavoidably postponed, will appear in our next.*

